# Pre-emptive and therapeutic adoptive immunotherapy for nasopharyngeal carcinoma: Phenotype and effector function of T cells impact on clinical response

**DOI:** 10.1080/2162402X.2016.1273311

**Published:** 2017-02-08

**Authors:** Corey Smith, Victor Lee, Andrea Schuessler, Leone Beagley, Sweera Rehan, Janice Tsang, Vivian Li, Randal Tiu, David Smith, Michelle A. Neller, Katherine K. Matthews, Emma Gostick, David A. Price, Jacqueline Burrows, Glen M. Boyle, Daniel Chua, Benedict Panizza, Sandro V. Porceddu, John Nicholls, Dora Kwong, Rajiv Khanna

**Affiliations:** a QIMR Berghofer Centre for Immunotherapy and Vaccine Development and Tumour Immunology Laboratory, Department of Immunology, QIMR Berghofer Medical Research Institute, Brisbane, Queensland, Australia; b Department of Clinical Oncology, Queen Mary Hospital, The University of Hong Kong, Hong Kong; c Institute of Infection and Immunity, Cardiff University School of Medicine, Cardiff, UK; d Vaccine Research Center, National Institute of Allergy and Infectious Diseases, National Institutes of Health, Bethesda, MD, USA; e Comprehensive Oncology Centre, Hong Kong Sanatorium Hospital, Hong Kong; f Department of Otolaryngology-Head and Neck Surgery, The Princess Alexandra Hospital, University of Queensland, Brisbane, Queensland, Australia; g Department of Pathology, Queen Mary Hospital, The University of Hong Kong, Hong Kong

**Keywords:** Adoptive immunotherapy, Epstein-Barr virus, T cells, safety, nasopharyngeal carcinoma

## Abstract

Adoptive T cell therapy has emerged as a powerful strategy to treat human cancers especially haematological malignancies. Extension of these therapies to solid cancers remains a significant challenge especially in the context of defining immunological correlates of clinical responses. Here we describe results from a clinical study investigating autologous Epstein-Barr virus (EBV)-specific T cells generated using a novel AdE1-LMPpoly vector to treat patients with nasopharyngeal carcinoma (NPC) either pre-emptively in at-risk patients with no or minimal residual disease (N/MRD) or therapeutically in patients with active recurrent/metastatic disease (ARMD). Tolerability, safety and efficacy, including progression-free survival (PFS) and overall survival (OS), were evaluated following adoptive T-cell immunotherapy. Twenty-nine patients, including 20 with ARMD and nine with N/MRD, successfully completed T-cell therapy. After a median follow-up of 18.5 months, the median PFS was 5.5 months (95% CI 2.1 to 9.0 months) and the median OS was 38.1 months (95% CI 17.2 months to not reached). Post-immunotherapy analyses revealed that disease stabilization in ARMD patients was significantly associated with the functional and phenotypic composition of *in vitro*-expanded T cell immunotherapy. These included a higher proportion of effector CD8^+^ T-cells and an increased number of EBV-specific T-cells with broader antigen specificity. These observations indicate that adoptive immunotherapy with AdE1-LMPpoly-expanded T cells stabilizes relapsed, refractory NPC without significant toxicity. Promising clinical outcomes in N/MRD patients further suggest a potential role for this approach as a consolidation treatment following first-line chemotherapy.

## Introduction

Epstein-Barr virus (EBV)-associated nasopharyngeal carcinoma (NPC) is endemic in South-East Asia^1^. Current estimates indicate that 80,000 new cases of EBV-associated NPC are diagnosed annually, with the highest incidence occurring in Southern China^2,3^. Precision radiotherapy is recommended for early-stage NPC while concurrent chemoradiotherapy with or without adjunct chemotherapy is the mainstay of treatment for locoregionally advanced disease^4-6^. In addition, plasma EBV DNA is considered the most accurate predictive and prognostic biomarker of disease diagnosis, treatment response and prognostication^7-11^. Despite intensive treatment, 30–40% of patients still develop locoregional relapse or distant failure. Local salvage operation with or without post-operative radio/chemotherapy or a second-course of radiotherapy with or without chemotherapy can attain a locoregional control rate between 20 and 100%^12^. However, salvage treatment is associated with significant long-term treatment-related toxicities including brain necrosis, nasopharyngeal mucosal necrosis, palatal fistula and massive bleeding. Although palliative chemotherapy can offer median progression-free survival (PFS) between 3 and 9 months for patients with recurrent or metastatic disease, the overall survival (OS) remains disappointing^13^. Novel therapeutic approaches are therefore required to reduce disease burden in these patients and ideally to pre-empt relapse in high-risk patients with no radiologically demonstrable disease, including those with substantial baseline and post-treatment levels of plasma EBV DNA^7-9,14^.

Although latent EBV infection is usually under the strict control of CD8^+^ cytotoxic T-lymphocytes in immunocompetent individuals, EBV-infected NPC cells are characterized by a restricted gene expression pattern, typically limited to EBV nuclear antigen (EBNA) 1, latent membrane protein (LMP) 1 and LMP2^15^. EBNA1 and LMP1&2 are subdominant antigens that induce low numbers of functionally constrained memory T-cells which may be readily more susceptible to tumor-mediated immune suppression^16, 17^. Immunotherapeutic approaches to improve the frequency and function of LMP/EBNA1-specific T-cells may therefore augment current treatment options for NPC^16-18^.

We previously reported the preliminary outcome of a phase I study investigating the use of LMP1&2 and EBNA1-specific CTL immunotherapy generated using a novel adenoviral vector, AdE1-LMPpoly, in a cohort of palliative NPC patients with active refractory disease^19^. In the current study, we provide an extended analysis of a large cohort of NPC patients, including pre-emptive and therapeutic treatment of patients with no or minimal residual disease (N/MRD) and with active recurrent/metastatic disease (ARMD) respectively. We demonstrate that AdE1-LMPpoly-based T-cell therapy was well tolerated in the majority of NPC patients with highly encouraging clinical responses in both pre-emptive and therapeutic settings. We further show how the phenotypic composition and antigen specificity of adoptively transferred T cells is associated with disease stabilization in ARMD patients.

## Results

### Patient characteristics

**Figure 1.**
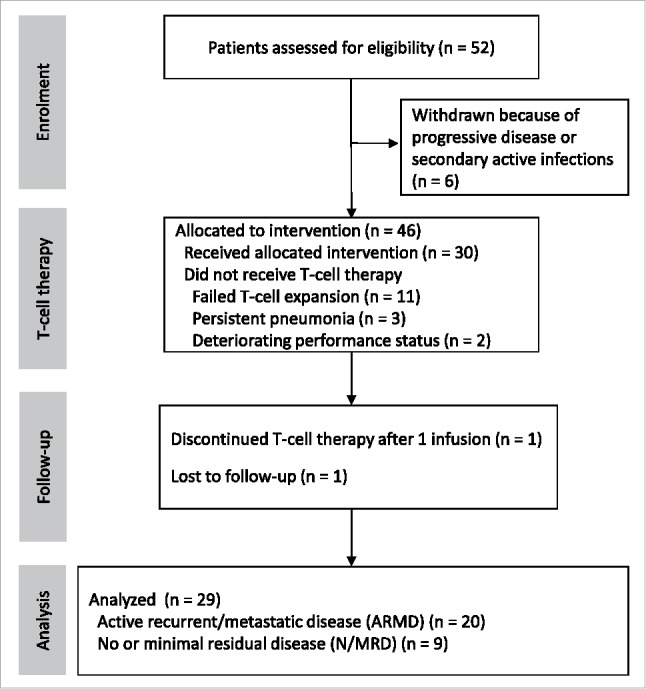
CONSORT diagram showing NPC patients recruitment, adoptive immunotherapy and clinical follow up.

Fifty-two patients were enrolled in this study as shown in the CONSORT diagram (Fig 1). Six patients were withdrawn prior to T-cell generation because of further PD after chemotherapy or secondary active infections. T-cell expansion was unsuccessful from 11 patients, due to either low specificity or poor yield. Another 5 patients were excluded from T-cell infusion because of persistent pneumonia (n = 3) or deteriorating ECOG performance status (2 or 3, n = 2). In total, 20 patients with ARMD and 9 patients with N/MRD received at least two doses of immunotherapy (range 2 to 6, median 4). The clinical characteristics of all patients who received adoptive T-cell therapy are shown in Table 1 and Data Supplement Table S1. Patient 33 died after the administration of a single dose because of a lung abscess complicating aspiration pneumonia.

**Table 1.** Clinical characteristics of NPC patients treated with AdE1-LMPpoly T-cells.

	ARMD	N/MRD
	(n = 21)	(n = 9)
Median age in years (range)	46 (34–68)	49 (22–66)
Sex		
Male	18	8
Female	2	1
Stage on diagnosis		
I	2	2
II	2	1
III	7	4
IVA	7	1
IVB	1	1
IVC	2	0
Median number of lines of chemotherapy before T-cell therapy (range)	3 (1 to 5)	2 (1 to 4)
History of recurrent NPC	21	7
Disease status at first T-cell infusion		
No radiological disease	0	9
Local recurrence	11	0
Regional nodal recurrence	6	0
Lung metastasis	9	0
Liver metastasis	5	0
Bone metastasis	5	0
Distant nodal metastasis	4	0
Median plasma EBV DNA copies/mLbefore T-cell therapy (range)	2.3 × 10^3^ (0 to 6.3 × 10^6^)	0 (0)

Abbreviations: ARMD, active recurrent/metastatic disease; EBV DNA, Epstein-Barr virus deoxyribonucleic acid; N/MRD, no or minimal residual disease; NPC, nasopharyngeal carcinoma.

### Functional and phenotypic properties of AdE1-LMPpoly expanded T-cells

**Figure 2.**
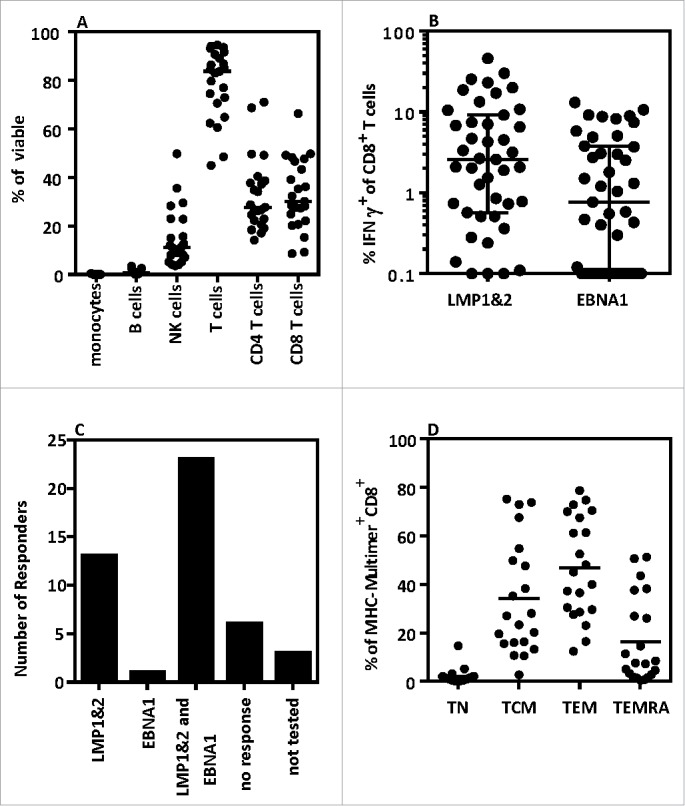
Functional and phenotypic characteristics of AdE1-LMPpoly-expanded T-cells (A) The phenotypic characteristics of AdE1-LMPpoly-stimulated T-cells were assessed by measuring the surface expression of CD14 (monocytes), CD19 (B cells), CD16 (NK cells), CD3 (T-cells), CD4 (CD4 T-cells) and CD8 (CD8 T cells). (B) AdE1-LMPpoly-stimulated T-cells were assessed for the intracellular production of IFN-γ following recall with pools of CD8^+^ T-cell epitopes derived from LMP1&2 or EBNA1. The data represent the frequencies of LMP1&2-specific or EBNA1-specific IFN-γ-producing T-cells in all patients. (C) The data represent the number of patients generating either LMP1&2-specific and/or EBNA1-specific T-cells. (D) The memory characteristics of MHC-multimer^+^ T-cells were determined by measuring the surface expression of CCR7, CD45RA, CD27, CD28 and CD57. T-cell phenotype was determined as follows: Naïve (T_N_) CD45RA^+^CCR7^+^; Central Memory (T_CM_) CD45RA^−^CD27^+/−^CD28^+^CD57^−^; Effector Memory (T_EM_) CD45RA^−^CD27^−/+^CD28^−^CD57^+/−^; Effector Memory RA (T_EMRA_) CD45RA^+^CD27^−/+^CD28^−/+^CD57^+^.

**Figure 3.**
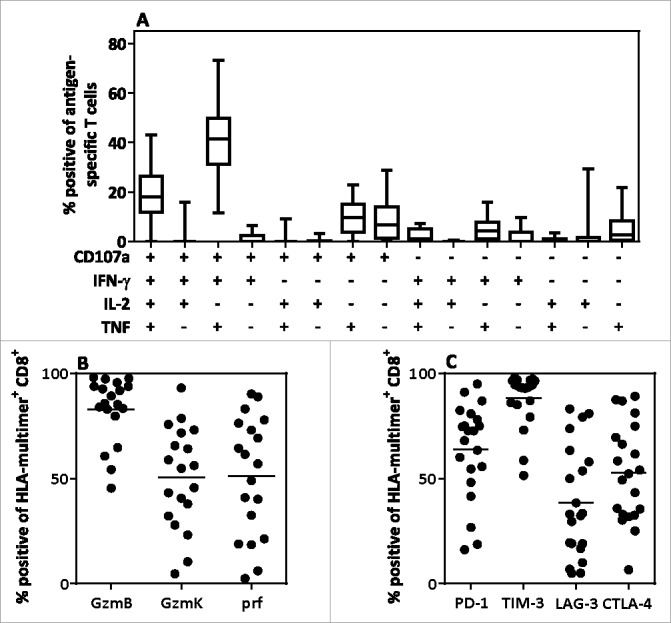
Polyfunctional cytokine profile and the expression of effector and co-inhibitory molecules in AdE1-LMPpoly-expanded T-cells. (A) AdE1-LMPpoly-stimulated T-cells were assessed for intracellular cytokine production (IFN-γ, TNF, IL-2) and degranulation (CD107a) following recall with pools of LMP/EBNA1-encoded CD8^+^ T-cell epitopes. The data represent the proportion of the total antigen-specific T-cells producing each combination of effector functions. (B, C) MHC-multimer^+^ CD8^+^ T-cells in the AdE1-LMPpoly T-cell products were assessed for intracellular expression of the cytolytic enzymes granzyme B (GzmB), granzyme K (GzmK) and perforin (Prf), or for surface expression of the co-inhibitory receptors PD-1, TIM-3, LAG-3 and CTLA-4. The data represent the proportion of MHC-multimer^+^ CD8^+^ T-cells expressing (B) effector molecules or (C) co-inhibitory receptors.

AdE1-LMPpoly-expanded products comprised a median of 83.80% CD3^+^ T-cells (Fig 2A). Of all mononuclear cells, 32.20% (median) were CD8^+^ T-cells and 27.70% (median) were CD4^+^ T-cells. NK cells comprised the majority of the remaining cells in the therapy. While the majority of patients had undetectable LMP1/2 and EBNA1-specific T cell ex vivo, twenty-three patients generated both LMP1/2-specific and EBNA1-specific T-cells post-expansion, 13 generated only LMP1/2-specific T-cells, 1 generated only EBNA1-specific T-cells, 6 failed to generate either LMP1/2-specific or EBNA1-specific T-cells, and 3 were not tested due to low cell numbers (Fig 2B & C). Two patients failed to generate enough T cells for infusion despite the presence of antigen-specific T cells. The majority of LMP/EBNA1 MHC-multimer^+^ CD8^+^ T-cells displayed either a central memory (TCM) or an effector memory (TEM) phenotype (Fig 2D). Most of these cells mobilized CD107a and expressed interferon (IFN)-γ and tumor necrosis factor (TNF) in response to antigen; a large proportion also co-expressed interleukin (IL)-2 (Fig. 3A). This polyfunctional profile was associated with high expression levels of the cytolytic enzymes granzyme B, granzyme K and perforin in LMP/EBNA1 MHC-multimer^+^ CD8^+^ T-cells (Fig. 3B). The checkpoint molecules PD-1, CTLA-4, LAG-3 and TIM-3 were also expressed on AdE1-LMPpoly-expanded LMP/EBNA1 MHC-multimer^+^ CD8^+^ T-cells (Fig. 3C). Univariable and multivariable analyses revealed that successful T-cell expansion was significantly correlated with age <55 years (*P* = .035), baseline white cell count before T-cell harvest (*P* = .015) and patients with N/MRD (*P* = .020) (Data Supplement Table S2).

### Safety evaluation

A total of 30 patients had at least 1 T-cell infusion and were included in the safety evaluation. None of these patients showed treatment-related grade 4 or grade 5 adverse events (Table 2). The majority of adverse events that could feasibly be attributed to T-cell infusion were classified as grade 1 or grade 2. These events were self-limiting and subsided spontaneously on the day after T-cell infusion without additional intervention. Two serious adverse events were recorded, possibly but not directly related to T-cell therapy. Independent review of the radiological, pathological and microbiological features associated with these cases showed that both patients had progressive tumor necrosis. This was potentially due to T-cell-induced inflammation and cytotoxicity but was more likely associated with complications of prior treatment for locally recurrent NPC. There was no evidence of microbial contamination in the AdE1-LMPpoly-expanded products.

**Table 2.** Safety profiles after T-cell therapy.

Adverse events	n = 30 (%)
**Grade 1**	10 (33.3%)
Fatigue	1 (3.3%)
Dry cough	1 (3.3%)
Fever	3 (10%)
Chills	1 (3.3%)
Chest pain	1 (3.3%)
Sore throat	1 (3.3%)
Hyperbilirubinemia	1 (3.3%)
Altered hearing ability	1 (3.3%)
**Grade 2**	6 (20%)
Fatigue	2 (6.7%)
Fever	1 (3.3%)
Dyspnea	1 (3.3%)
Headache	1 (3.3%)
Vomiting	1 (3.3%)
**Grade 3**	2 (6.7%)
Lung abscess	2 (6.7%)

### Clinical outcome of T-cell therapy in ARMD and N/MRD patients

**Figure 4.**
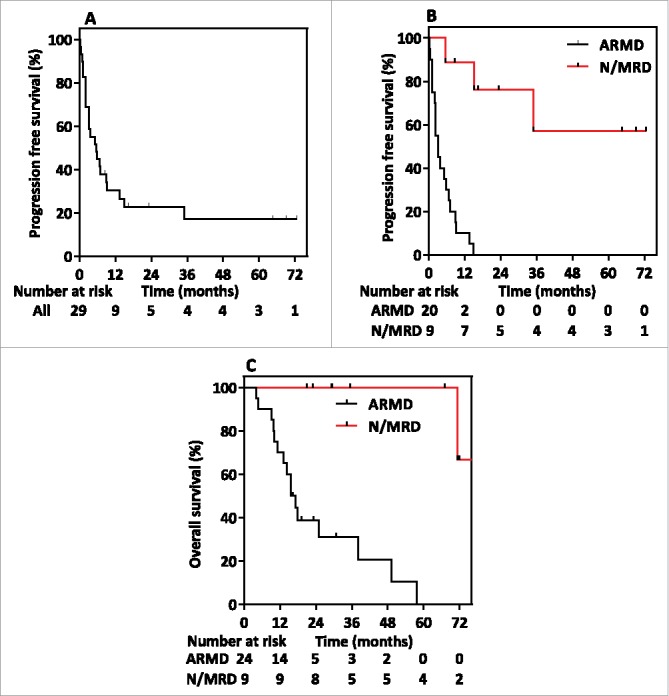
Kaplan-Meier curves showing (A) progression-free survival in the whole study population, (B) progression-free survival stratified by active recurrent/metastatic disease (ARMD) and no or minimal residual disease (N/MRD), and (C) overall survival from the time of recruitment stratified by ARMD and N/MRD.

The median overall PFS of NPC patients who completed T-cell therapy was 5.5 months (95% CI 2.1 to 9.0 months), and the corresponding 1-year and 2-year PFS rates were 29.4% and 21.5%, respectively (Fig 4A). The median overall survival (OS) of NPC patients who completed T-cell therapy was 38.1 months (95% CI 17.2 months to not reached), and the corresponding 1-year and 2-year OS rates were 72.4% and 41.3%, respectively. The disease control rate in the ARMD group was 60% (12 of 20 patients had stable disease following adoptive therapy). None of the ARMD patients developed a radiologically confirmed complete or partial response. Six N/MRD patients maintained a continuous molecular and radiographic complete response and were censored for progression analysis. One N/MRD patient developed PD, and another 2 N/MRD patients were initially stable and then progressed. The median PFS was significantly longer for N/MRD patients (not reached) compared with ARMD patients (3.2 months, 95% CI 0.4 to 3.7 months, *P* < .001) (Fig 4B). Univariable and multivariable analyses revealed that N/MRD status (hazard ratio, 95% CI 0.03 to 0.42, *P* = .002, Table S3) was an independent prognostic factor for PFS. Furthermore, the median OS was significantly longer for N/MRD patients (not reached, range 21 to 76.5 months) compared with ARMD patients (15.7 months, range 4.1 to 57.8 months, *P* < .001) (Fig. 4C). Eight N/MRD patients (88.9%) were alive at the conclusion of the study. Ten patients in the ARMD group and 3 patients in the N/MRD group received post-T-cell infusion chemotherapy. As reported previously, patient 11 developed significant tumor shrinkage and an excellent partial response to paclitaxel salvage chemotherapy following the administration of AdE1-LMPpoly-expanded T-cells.^19^

### AdE1-LMPpoly-generated T-cell correlates of clinical response

**Figure 5.**
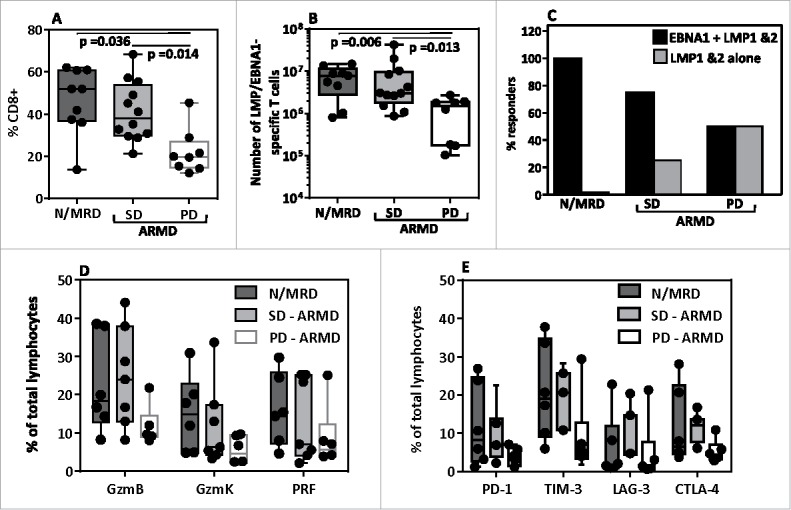
Impact of phenotype and antigen specificity of administered T-cells on the clinical response to adoptive immunotherapy. (A) The frequency of CD8^+^ T-cells in the AdE1-LMPpoly-expanded product administered to N/MRD and ARMD (stable disease [SD] *vs* progressive disease [PD]) patients. (B) The total number of LMP/EBNA1-specific T-cells infused into N/MRD and ARMD (SD *vs* PD) patients. (C) The percentage of N/MRD and ARMD (SD *vs* PD) patients who received a product containing CD8^+^ T-cells specific for either LMP1&2 and EBNA1 or LMP1&2 alone. (D) The percentage of T-cells expressing granzyme B (GzmB), granzyme K (GzmK) or perforin (Prf) in the total lymphocyte population administered to N/MRD and ARMD (SD *vs* PD) patients. (E) The percentage of T-cells expressing PD-1, TIM-3, LAG-3 or CTLA-4 in the total lymphocyte population administered to N/MRD and ARMD (SD *vs* PD) patients.

The identification of immune correlates that predict disease control remains a significant challenge, especially in the field of solid tumors. Quantitative analysis of circulating LMP/EBNA1-specific T-cell responses in ARMD patients following adoptive immunotherapy revealed no significant correlations with outcome (data not shown). To explore other correlates we compared survival, and T cell phenotype and specificity in ARMD patients who had SD or PD. ARMD patients with SD had a median PFS of 5.5 months and OS of 18 months, while ARMD patients with PD had a median PFS of 1 month and OS of 13.5 months. We noted that the proportion of CD8^+^ T-cells in the administered AdE1-LMPpoly-expanded product was significantly lower for ARMD patients with PD compared with ARMD patients with SD (Fig 5A). Interestingly, both ARMD patients with SD and N/MRD patients showed a comparable proportion of CD8^+^ T cells. Similarly, we noted that ARMD patients with SD and N/MRD patients received a significantly higher number of LMP/EBNA1-specific T-cells compared with ARMD patients with PD (Fig 5B). The AdE1-LMPpoly-expanded products from ARMD patients with SD and N/MRD patients were also more likely to contain T-cells that recognized both LMP1&2 and EBNA1 compared with those from ARMD patients with PD (Fig 5C). We also assessed the functional and phenotypic attributes of the CD8^+^ T-cells administered to ARMD patients. Although not significant, we found that disease stabilization was associated with a trend towards increased numbers of effector CD8^+^ T-cells (Fig 5D). A similar trend between groups was observed for CD8^+^ T-cells expressing checkpoint molecules, most notably CTLA-4 (Fig 5E). There was no correlation between either pre-treatment or post-treatment plasma EBV DNA load and PFS in patients with ARMD (data not shown). Collectively, these analyses suggest that qualitative aspects of the T-cell infusate may influence immunotherapeutic outcomes in patients with NPC.

## Discussion

NPC remains a significant problem in endemic regions of Southeast Asia. However, the defined association with EBV infection provides a platform to develop virus-specific therapeutic approaches^20-25^. In this study, we explored the use of an adoptive cellular therapy targeting the LMP1&2 and EBNA1 antigens expressed in NPC. The AdE1-LMPpoly vector rapidly expanded T-cells with improved functional and cytolytic potential compared with EBV-specific T-cells manufactured previously using a different approach based on stimulation with lymphoblastoid cell lines (LCLs) stimulation^26^. These autologous AdE1-LMPpoly-expanded T cells were associated predominantly with self-limiting grade 1 and grade 2 adverse events in NPC patients. Lung abscesses were recorded as grade 3 events in 2 of 30 patients, but most likely represented complications of prior treatment for locally recurrent NPC. Clinical benefit was also observed after the adminstration of AdE1-LMPpoly-expanded T-cells, objectified by disease stabilization in ARMD patients and remarkably long (not yet defined) PFS and OS in N/MRD patients.

The AdE1-LMPpoly vector was designed to promote the optimal expansion of LMP/EBNA1-specific T-cells from low frequency precursors^17,28^. Consistent with our previous observations, LMP/EBNA1-specific T-cells could be generated from the majority of NPC patients. This contrasts with the use of EBV-transformed LCLs, which stimulate detectable LMP-specific T-cells in only half of all cellular products^21,24,28,^. Recent studies using LCL-generated products have shown the potential benefit of LMP-specific T-cells in the treatment of NPC. Objective clinical responses including both complete and partial responses have been detected in patients following T cell therapy and the presence of LMP2-specific T cells has been associated with improved clinical responses when T cells were delivered as a consolidative treatment to standard chemotherapy^24,26,28^. While differences in patient cohorts may account for differences in responses in the current study and previous studies, the capacity to optimise LMP/EBNA1-specific T-cell numbers with the AdE1-LMPpoly vector should provide a platform to further improve clinical outcome in NPC patients. In line with this possibility, we found evidence for an association between disease stabilization and the number of LMP/EBNA1-specific T-cells administered to ARMD patients. Future optimization of LMP/EBNA-1-specific T-cell frequencies, potentially via the use of allogeneic “off-the-shelf” products, may further enhance the quality and efficacy of adoptive T-cell therapies^29-31^.

One approach that could potentially improve the therapeutic benefit of adoptive immunotherapy in NPC patients is its application in patients with minimal residual disease who are at a high risk of relapse. Recent observations have shown that patients with residual plasma EBV DNA titers after standard radio/chemotherapy are at much higher risk of relapse^32,33^. In the current study, we found that LMP/EBNA1-specific T-cells were generated more consistently from N/MRD patients. Early intervention may therefore promote greater T-cell expansions compared with identical approaches in highly pre-treated patients with refractory disease. This notion is supported by the observation that plasma EBV DNA was undetectable at baseline in the present cohort of N/MRD patients.

Significant functional and phenotypic differences were apparent in the cellular products generated from NPC patients following AdE1-LMPpoly-based T-cell stimulation. Furthermore, we detected associations between disease stabilization and the number of LMP/EBNA1-specific T-cells, the proportion of CD8^+^ T-cells and the expression of effector molecules in the adoptively transferred infusate. Checkpoint molecules were also upregulated on the expanded LMP/EBNA1-specific T-cells. Although the role of immunoregulatory molecules has not been fully elucidated in this setting, clinical observations suggest that PD-L1 is expressed on NPC cells and may be associated with poor outcome^34^. Given the observed expression of PD-1 on the majority of LMP/EBNA1-specific T-cells, these findings suggest that PD-1:PD-L1 blockade could potentiate the efficacy of EBV-specific immunotherapy in NPC patients^35^.

In conclusion, the current study demonstrates that adoptive immunotherapy with AdE1-LMPpoly-stimulated T-cells is well tolerated in ARMD and N/MRD patients. Our results have also paved the way for an ongoing phase I/II study investigating the safety and efficacy of adoptive T-cell transfer as consolidation therapy in patients with minimal residual disease following first-line chemotherapy for metastatic NPC (ACTRN12613000866707).

## Materials and methods

### Patient recruitment

This prospective study was conducted according to the principles of the Declaration of Helsinki and approved by the QIMR Berghofer Medical Research Institute Human Research Ethics Committee, the Institutional Review Board of The University of Hong Kong/Hospital Authority Hong Kong West Cluster and the Metro South Human Research Ethics Committee before commencement. This study was registered under the Australia New Zealand Clinical Trial Registry (ACTRN12609000675224). Two cohorts of NPC patients were recruited from January 2008 to December 2015. The first cohort included 41 ARMD patients with histologically and/or radiologically confirmed local, regional or distant relapse of NPC after primary radical radiotherapy/chemoradiotherapy. Most of these patients had been treated with at least one line of palliative chemotherapy and/or surgery. The second cohort of 11 N/MRD patients received adoptive T-cell therapy while in disease remission after primary treatment or successful salvage treatment for locoregional recurrence or oligometastasis. Written informed consent was obtained in all cases. Baseline staging investigations included magnetic resonance imaging with T1, T2 and T1-gadolinum enhanced sequences of the head and neck or computed tomography (CT) scans with contrast of the head and neck, chest and abdomen before peripheral blood T-cell harvest, therapy manufacture, and infusion. Plasma EBV DNA was also measured at baseline. Disease stage at initial diagnosis was classified according to the American Joint Committee on Cancer staging manual 7th edition.

### Manufacture and adoptive transfer of LMP/EBNA1-specific T-cells

The clinical grade AdE1-LMPpoly vector used in this study has been described previously^19^. The AdE1-LMpoly vector contains a polyepitope of 16 HLA-restricted LMP1&2 epitopes fused to a truncated gly/ala deleted EBNA1 gene^36^. To generate LMP/EBNA1-specific T-cells, peripheral blood mononuclear cells (PBMCs) were first harvested from 100–300 mL of venous blood. The AdE1-LMPpoly vector was then used to infect 30% of the PBMCs (MOI of 10:1), which were then irradiated and co-cultured with the remaining PBMCs for two weeks. Cultures were supplemented with fresh growth medium and 120 IU/mL of recombinant IL-2 every 3–4 days (Komtur Pharmaceuticals). Expanded T-cells were tested for antigen specificity and microbial contamination prior to release for infusion. A summary of the number of infusions and the absolute number of transferred T-cells is shown in Table 1 and Table S1.

### Intracellular cytokine assay

For analysis of LMP1&2-specific and EBNA1-specific T-cell frequencies, AdE1-LMPpoly-expanded products were stimulated for 4 hours in the presence of GolgiPlug (BD Biosciences) with a pool of defined epitopes from LMP1&2 or EBNA1 or with an overlapping set of peptides encompassing the whole EBNA1 protein (all from Mimotopes, GenScript or JPT Technologies), and then assessed for the intracellular production of IFN-γ. For multiparametric analysis, cells were stimulated for 4 hours in the presence of GolgiPlug and GolgiStop (BD Biosciences) with the peptides listed above and anti-CD107a-FITC (BD Biosciences). Cells were then washed and stained with anti-CD8-PerCPCy5.5 (eBioscience) and anti-CD4-PECy7 (BD Biosciences), fixed and permeabilised with Cytofix/Cytoperm (BD Biosciences), washed again and stained with anti-IFN-γ-AF700, anti-IL-2-PE and anti-TNF-APC (all from BD Biosciences). After a further wash, cells were resuspended in PBS and acquired using a BD LSR Fortessa with FACSDiva software (BD Biosciences). Post-acquisition and Boolean analysis was performed using FlowJo software (TreeStar).

### Polychromatic profiling of T-cell phenotype

T-cells were incubated for 20 minutes at 4°C with APC-labelled MHC class I multimers specific for the HLA-A11-restricted epitope SSCSSCPLSKI (LMP2A), the HLA-A24-restricted epitope TYGPVFMCL (LMP2A) or the HLA-Cw03-restricted epitope FVYGGSKTSL (EBNA1). For assessment of surface phenotype, cells were then incubated for a further 30 minutes at 4°C with the following antibody panels: (i) anti-CD3-AF700, anti-CD4-PECy7 and anti-CD8-V500; (ii) anti-CD4-AF700, anti-CD8-V500, anti-CD14-eFluor450, anti-CD19-eFluor450 and anti-CD56-PECy7; (iii) anti-CD4-PECy7, anti-CD8-V500, anti-CD27-PE, anti-CD28-PerCPCy5.5, anti-CD45RA-FITC, anti-CCR7-AF700 and anti-CD57-biotin followed by steptavidin conjugated to Cascade Yellow; or (iv) anti-CD4-PECy7, anti-CD8-V500, anti-TIM-3-PE, anti-LAG-3-FITC and anti-PD-1-BV786. For intracellular analysis, cells were treated with TF Fixation/Permeabilization buffer (BD Biosciences) and then stained in the presence Perm/Wash with the following antibodies: (i) anti-perforin-BV421, anti-granzyme B-AF700 and anti-granzyme K-FITC; or (ii) anti-CTLA-4-BV421. Cells were acquired using a BD LSR Fortessa with FACSDiva software (BD Biosciences) and post-acquisition analysis was performed using FlowJo software (TreeStar).

### Clinical response evaluation

Patients with successful T-cell expansion and satisfactory Eastern Cooperative Oncology Group (ECOG) performance status (0 or 1) received a minimum of 2 infusions at fortnightly intervals and a total dose of between 4.9 × 10^7^ and 2.4 × 10^8^ AdE1-LMPpoly-expanded T-cells. One patient who received a single T-cell infusion was included only in the safety analysis. Patients were monitored fortnightly for tolerability and safety. MRI with T1, T2 and T1-gadolinum-enhanced sequence or CT scans with contrast of the head and neck were performed at 1, 2, 3, 4 and 6 months after first T-cell infusion and then every 3 months until progressive disease (PD), as we previously described.^19^ Surveillance CT scans with contrast of the thorax and abdomen were also performed every 3 months until PD. Acute and long-term toxicities were graded according to version 3.0 of the Common Terminology Criteria for Adverse Events (CTCAE).^37^ Best objective responses were determined according to version 1.1 of the Response Evaluation Criteria in Solid Tumors (RECIST).^38^

### Statistical analysis

PFS was calculated from the date of first T-cell infusion to the date of radiologically documented PD or the date of death. Patients who were alive and without PD were censored at their last follow-up. Mann-Whitney U tests were used to compare means between groups when there was evidence of deviation from normality assumptions. Log-rank tests were employed for subgroup comparisons of PFS. Binary logistic regressions with univariable and multivariable analyses were performed for factors correlated with successful T-cell expansion. Cox proportional hazard models with univariable and multivariable analyses were performed for prognostic factors of PFS. Data for plasma EBV DNA titers were log-transformed and analyzed on the log scale longitudinally. Time-dependent covariate Cox models were applied when considering the effect of log plasma EBV DNA titers and fold change in plasma EBV DNA levels from baseline. Statistical significance was defined as *P* < .05 (two-sided), and adjustment for multiple comparisons among group means was based on the Sidak method. All statistical analyses were performed using Prism 6 Software (GraphPad), Stata 13 (StataCorp LP), and Statistical Package for Social Sciences (SPSS) version 23.

## Supplementary Material

KONI_A_1273311_s02.docx

## References

[1] Ho JHC Nasopharyngeal carcinoma (NPC). Advances in Cancer Research 1972; 16:57-92; PMID:4333791 10.1016/s0065-230x(08)60372-3 4333791

[2] Zeng Y Seroepidemiological studies on nasopharyngeal carcinoma in China. Advances in Cancer Research 1985; 44:121-38; PMID:2994402 2994402 10.1016/s0065-230x(08)60027-5

[3] Senba M, Zhong XY, Senba MI, Itakura H EBV and nasopharyngeal carcinoma. Lancet 1994; 343:1104; PMID:7909127 10.1016/s0140-6736(94)90217-8 7909127

[4] Blanchard P, Lee A, Marguet S, Leclercq J, Ng WT, Ma J et al. Chemotherapy and radiotherapy in nasopharyngeal carcinoma: an update of the MAC-NPC meta-analysis. Lancet Oncol 2015; 16:645-55 25957714 10.1016/S1470-2045(15)70126-9

[5] Lee AW, Ma BB, Ng WT, Chan AT Management of Nasopharyngeal Carcinoma: Current Practice and Future Perspective. J Clin Oncol 2015; 33:3356-64 26351355 10.1200/JCO.2015.60.9347

[6] Sze H, Blanchard P, Ng WT, Pignon JP, Lee AW Chemotherapy for Nasopharyngeal Carcinoma - Current Recommendation and Controversies. Hematology/oncology clinics of North America 2015; 29:1107-22 26568551 10.1016/j.hoc.2015.07.004

[7] Chan AT, Lo YM, Zee B, Chan LY, Ma BB, Leung SF et al. Plasma Epstein-Barr virus DNA and residual disease after radiotherapy for undifferentiated nasopharyngeal carcinoma. J Natl Cancer Inst 2002; 94:1614-9 12419787 10.1093/jnci/94.21.1614

[8] Leung SF, Chan KC, Ma BB, Hui EP, Mo F, Chow KC et al. Plasma Epstein-Barr viral DNA load at midpoint of radiotherapy course predicts outcome in advanced-stage nasopharyngeal carcinoma. Ann Oncol 2014; 25:1204-8 24638904 10.1093/annonc/mdu117

[9] Leung SF, Zee B, Ma BB, Hui EP, Mo F, Lai M et al. Plasma Epstein-Barr viral deoxyribonucleic acid quantitation complements tumor-node-metastasis staging prognostication in nasopharyngeal carcinoma. J Clin Oncol 2006; 24:5414-8 17135642 10.1200/JCO.2006.07.7982

[10] Lin JC, Wang WY, Chen KY, Wei YH, Liang WM, Jan JS et al. Quantification of plasma Epstein-Barr virus DNA in patients with advanced nasopharyngeal carcinoma. N Engl J Med 2004; 350:2461-70 15190138 10.1056/NEJMoa032260

[11] Lo YM, Chan LY, Lo KW, Leung SF, Zhang J, Chan AT et al. Quantitative analysis of cell-free Epstein-Barr virus DNA in plasma of patients with nasopharyngeal carcinoma. Cancer Res 1999; 59:1188-91 10096545

[12] Wei WI, Kwong DL Recurrent nasopharyngeal carcinoma: surgical salvage vs. additional chemoradiation. Current opinion in otolaryngology & head and neck surgery 2011; 19:82-6; PMID:21412154; http://dx.doi.org/10.1097/MOO.0b013e328344a599 21412154

[13] Chan OS, Ngan RK Individualized treatment in stage IVC nasopharyngeal carcinoma. Oral Oncol 2014; 50:791-7 24518920 10.1016/j.oraloncology.2014.01.004

[14] Leonard WJ The defective gene in X-linked severe combined immunodeficiency encodes a shared interleukin receptor subunit: implications for cytokine pleiotropy and redundancy. Current Opinion in Immunology 1994; 6:631-5; PMID:7946053 7946053 10.1016/0952-7915(94)90152-x

[15] Nicholls J, Hahn P, Kremmer E, Fröhlich T, Arnold GJ, Sham J et al. Detection of wild type and deleted latent membrane protein 1 (LMP1) of Epstein-Barr virus in clinical biopsy material. Journal of virological methods 2004; 116:79-88 14715310 10.1016/j.jviromet.2003.10.015

[16] Smith C, and Khanna R. Polyepitope Vaccines for Human Cancers Hoboken, NJ: John Wiley and Sons, Inc., 2007.

[17] Smith C, Cooper L, Burgess M, Rist M, Webb N, Lambley E et al. Functional reversion of antigen-specific CD8+ T cells from patients with Hodgkin lymphoma following in vitro stimulation with recombinant polyepitope. The Journal of Immunology 2006; 177:4897-906 16982932 10.4049/jimmunol.177.7.4897

[18] Smith C, Khanna R Generation of cytotoxic T lymphocytes for immunotherapy of EBV-associated malignancies. Methods in Molecular Biology (Clifton, NJ) 2010; 651:49-59 10.1007/978-1-60761-786-0_3 20686959

[19] Smith C, Tsang J, Beagley L, Chua D, Lee V, Li V et al. Effective treatment of metastatic forms of Epstein-Barr virus-associated nasopharyngeal carcinoma with a novel adenovirus-based adoptive immunotherapy. Cancer Res 2012; 72:1116-25 22282657 10.1158/0008-5472.CAN-11-3399

[20] Comoli P, De Palma R, Siena S, Nocera A, Basso S, Del Galdo F et al. Adoptive transfer of allogeneic Epstein-Barr virus (EBV)-specific cytotoxic T cells with in vitro antitumor activity boosts LMP2-specific immune response in a patient with EBV-related nasopharyngeal carcinoma. Ann Oncol 2004; 15:113-7 14679129 10.1093/annonc/mdh027

[21] Gottschalk S, Heslop HE, Rooney CM Adoptive immunotherapy for EBV-associated malignancies. Leuk Lymphoma 2005; 46:1-10 15621775 10.1080/10428190400002202

[22] Lutzky VP, Corban M, Heslop L, Morrison LE, Crooks P, Hall DF et al. Novel approach to the formulation of an Epstein-Barr virus antigen-based nasopharyngeal carcinoma vaccine. J Virol 2010; 84:407-17 19846527 10.1128/JVI.01303-09 PMC2798422

[23] Moss D, Khanna R, Bharadwaj M Will a vaccine to nasopharyngeal carcinoma retain orphan status? Developments in biologicals 2002; 110:67; PMID:12477308 12477308

[24] Straathof KC, Bollard CM, Popat U, Huls MH, Lopez T, Morriss MC et al. Treatment of nasopharyngeal carcinoma with Epstein-Barr virus–specific T lymphocytes. Blood 2005; 105:1898-904 15542583 10.1182/blood-2004-07-2975

[25] Yufeng D, Guocheng Z, Dongliang X, Rong F, Yuhong C, Ruying L et al. Whole-tumor-antigen-pulsed dendritic cells elicit cytotoxic T-cell response against pediatric nasopharyngeal carcinoma in vitro. Medical oncology 2009; 26:78-85 18810669 10.1007/s12032-008-9093-8

[26] Louis CU, Straathof K, Bollard CM, Ennamuri S, Gerken C, Lopez TT et al. Adoptive transfer of EBV-specific T cells results in sustained clinical responses in patients with locoregional nasopharyngeal carcinoma. J Immunother 2010; 33:983-90 20948438 10.1097/CJI.0b013e3181f3cbf4 PMC2964409

[27] Smith C, Beagley L, Khanna R Acquisition of polyfunctionality by Epstein-Barr virus-specific CD8+ T cells correlates with increased resistance to galectin-1-mediated suppression. J Virol 2009; 83:6192-8 19357166 10.1128/JVI.00239-09 PMC2687380

[28] Chia WK, Teo M, Wang WW, Lee B, Ang SF, Tai WM et al. Adoptive T-cell transfer and chemotherapy in the first-line treatment of metastatic and/or locally recurrent nasopharyngeal carcinoma. Mol Ther 2014; 22:132-9 24297049 10.1038/mt.2013.242 PMC3978790

[29] Barker JN, Doubrovina E, Sauter C, Jaroscak JJ, Perales MA, Doubrovin M et al. Successful treatment of EBV-associated posttransplantation lymphoma after cord blood transplantation using third-party EBV-specific cytotoxic T lymphocytes. Blood 2010; 116:5045-9 20826724 10.1182/blood-2010-04-281873 PMC3012598

[30] Leen AM, Bollard CM, Mendizabal AM, Shpall EJ, Szabolcs P, Antin JH et al. Multicenter study of banked third-party virus-specific T cells to treat severe viral infections after hematopoietic stem cell transplantation. Blood 2013; 121:5113-23 23610374 10.1182/blood-2013-02-486324 PMC3695359

[31] Melenhorst JJ, Castillo P, Hanley PJ, Keller MD, Krance RA, Margolin J et al. Graft versus leukemia response without graft-versus-host disease elicited by adoptively transferred multivirus-specific T-cells. Mol Ther 2015; 23:179-83 25266309 10.1038/mt.2014.192 PMC4426803

[32] Hsu CL, Chang KP, Lin CY, Chang HK, Wang CH, Lin TL et al. Plasma Epstein-Barr virus DNA concentration and clearance rate as novel prognostic factors for metastatic nasopharyngeal carcinoma. Head Neck 2012; 34:1064-70 22083949 10.1002/hed.21890

[33] Wang WY, Twu CW, Chen HH, Jan JS, Jiang RS, Chao JY et al. Plasma EBV DNA clearance rate as a novel prognostic marker for metastatic/recurrent nasopharyngeal carcinoma. Clin Cancer Res 2010; 16:1016-24 20103659 10.1158/1078-0432.CCR-09-2796

[34] Zhang J, Fang W, Qin T, Yang Y, Hong S, Liang W et al. Co-expression of PD-1 and PD-L1 predicts poor outcome in nasopharyngeal carcinoma. Medical oncology 2015; 32:86 25702326 10.1007/s12032-015-0501-6

[35] Fang W, Zhang J, Hong S, Zhan J, Chen N, Qin T et al. EBV-driven LMP1 and IFN-gamma up-regulate PD-L1 in nasopharyngeal carcinoma: Implications for oncotargeted therapy. Oncotarget 2014; 5:12189-202 25361008 10.18632/oncotarget.2608 PMC4322961

[36] Smith C, Cooper L, Burgess M, Rist M, Webb N, Lambley E et al. Functional reversion of antigen-specific CD8+ T cells from patients with Hodgkin lymphoma following in vitro stimulation with recombinant polyepitope. J Immunol 2006; 177:4897-906 16982932 10.4049/jimmunol.177.7.4897

[37] Cancer Therapy Evaluation Program, Common Terminology Criteria for Adverse Events (CTCAE) v4.0 National Cancer Institute (NIH) USA, 2016:http://ctep.cancer.gov/protocolDevelopment/electronic_applications/ctc.htm

[38] Eisenhauer EA, Therasse P, Bogaerts J, Schwartz LH, Sargent D, Ford R et al. New response evaluation criteria in solid tumours: revised RECIST guideline (version 1.1). Eur J Cancer 2009; 45:228-47 19097774 10.1016/j.ejca.2008.10.026

