# Supplementary material for: Pre-emptive and therapeutic adoptive immunotherapy for nasopharyngeal carcinoma: Phenotype and effector function of T cells impact on clinical response
Source: Oncoimmunology. 2017 Feb 8;6(2):e1273311. doi: 10.1080/2162402X.2016.1273311 (PMC5353921; doi:10.1080/2162402X.2016.1273311)
Supplement: KONI_A_1273311_s02.docx [file koni-06-02-1273311-s001.docx]

**SUPPLEMENTAL TABLES**

**Table S1:** Clinical Characteristics of All Patients Treated with AdE1-LMpoly T-Cells

| **Patient Code** | **Age** | **Sex** | | **Stage at diagnosis** | | **Site of recurrence** | | **Disease status at the time of T-cell infusion** | **Plasma EBV DNA at T-cell infusion (copies/ml)** | **Number of lines of pre-T-cell radio-therapy** | **Number of lines of pre-T-cell chemo-therapy** | **Number of T-cell infusions** | **Total T-cell dose (cell number)** | **Best objective response to T-cell therapy** | **PFS**  **(months)** | **OS from recruitment (months)** |
| --- | --- | --- | --- | --- | --- | --- | --- | --- | --- | --- | --- | --- | --- | --- | --- | --- |
| 2 | 44 | | M | | I (T1N0M0) | | Local | ARMD | 1.93 × 10^4^ | 3 | 3 | 5 | 1.20 × 10^8^ | SD | 13.5 | 17.2 |
| 3 | 42 | | M | | IVA (T4N0M0) | | Local, liver, lung | ARMD | 6.31 × 10^6^ | 2 | 3 | 3 | 6.10 × 10^7^ | SD | 5.8 | 9.8 |
| 5 | 42 | | M | | III (T3N1M0) | | Bone, liver | ARMD | 1.11 × 10^5^ | 3 | 2 | 4 | 9.20 × 10^7^ | SD | 2.0 | Alive* |
| 8 | 60 | | M | | IVC (T3N3bM1) | | Bone, lung | ARMD | 8.63 × 10^2^ | 2 | 3 | 5 | 1.10 × 10^8^ | SD | 3.7 | 17.8 |
| 9 | 50 | | M | | III (T2N2M0) | | Regional nodes, distant nodes | ARMD | 1.15 × 10^6^ | 1 | 4 | 3 | 7.20 × 10^7^ | PD | 1.1 | 10.1 |
| 10 | 34 | | M | | IVC (T4N3bM1) | | Regional nodes, bone | ARMD | 0 | 2 | 2 | 5 | 1.10 × 10^8^ | SD | 7.0 | 57.8 |
| 11 | 50 | | M | | II (T1N1M0) | | Local, regional nodes | ARMD | 2.28 × 10^4^ | 3 | 3 | 4 | 8.30 × 10^7^ | SD | 2.1 | 15.7 |
| 13 | 46 | | M | | I (T1N0M0) | | Local, lung | ARMD | 3.77 × 10^1^ | 1 | 1 | 4 | 9.20 × 10^7^ | SD | 9.1 | 38.1 |
| 14 | 53 | | M | | IVA (T4N2M0) | | Bone, liver, lung, distant nodes | ARMD | 1.20 × 10^6^ | 3 | 4 | 3 | 6.60 × 10^7^ | PD | 1.1 | 13.1 |
| 16 | 52 | | M | | IVB (T2N3M0) | | Local, regional nodes, lung, distant nodes | ARMD | 3.35 × 10^3^ | 1 | 3 | 4 | 9.20 × 10^7^ | SD | 15.0 | 25.0 |
| 17 | 50 | | M | | II (T2N1M0) | | Local | ARMD | 7.66 × 10^2^ | 2 | 4 | 3 | 6.10 × 10^7^ | SD | 2.1 | 4.8 |
| 20 | 42 | | M | | III (T2N2M0) | | Bone, liver, distant nodes | ARMD | 5.95 × 10^2^ | 4 | 4 | 6 | 1.60 × 10^9^ | PD | 2.1 | 14.3 |
| 23 | 44 | | F | | III (T3N1M0) | | Local, regional nodes | ARMD | 1.27 × 10^3^ | 2 | 2 | 8 | 2.40 × 10^8^ | SD | 5.1 | 49.3 |
| 24 | 37 | | M | | III (T2N2M0) | | Local | ARMD | 1.52 × 10^3^ | 2 | 2 | 3 | 8.40 × 10^7^ | PD | 1.0 | 15.7 |
| 27 | 39 | | M | | III (T2N2M0) | | Lung | ARMD | 1.21 × 10^4^ | 1 | 2 | 2 | 5.70 × 10^7^ | PD | 6.7 | Alive |
| 28 | 45 | | M | | III (T3N2M0) | | Lung | ARMD | 1.87 × 10^2^ | 2 | 5 | 6 | 1.30 × 10^8^ | PD | 3.2 | 9.2 |
| 33 | 60 | | M | | IVA (T4N1M0) | | Local | ARMD | 3.21 × 10^3^ | 2 | 6 | 1 | 2.71 × 10^7^ | NA | NA | NA |
| 101 | 66 | | M | | IVA (T4N2M0) | | Local, regional nodes | ARMD | 8.19 × 10^2^ | 2 | 2 | 6 | 1.80 × 10^8^ | SD | 9.0 | 11.1 |
| 109 | 68 | | M | | IVA (T4N1M0) | | Liver, lung | ARMD | 3.16 × 10^3^ | 1 | 3 | 5 | 1.30 × 10^8^ | SD | 3.2 | 16.3 |
| 112 | 41 | | M | | IVA (T4N1M0) | | Local | ARMD | 9.00 × 10^0^ | 1 | 2 | 4 | 1.10 × 10^8^ | PD | 0.5 | Alive |
| 113 | 54 | | F | | IVA (T4N0M0) | | Lung | ARMD | 6.81 × 10^4^ | 2 | 3 | 2 | 4.90 × 10^7^ | PD | 0.2 | 4.1 |
| 18 | 62 | | M | | I (T1N0M0) | | Local | N/MRD | 0 | 2 | 1 | 6 | 1.70 × 10^8^ | No progression | Censored | Alive |
| 26 | 40 | | M | | III (T2N2M0) | | Local, adrenal, bone | N/MRD | 0 | 3 | 4 | 6 | 1.7 × 10^8^ | No progression | Censored | Alive |
| 30 | 58 | | M | | I (T1N0M0) | | Local | N/MRD | 0 | 2 | 1 | 4 | 9.9 × 10^7^ | SD | 15.2 | Alive |
| 31 | 49 | | M | | III (T3N1M0) | | Local | N/MRD | 0 | 2 | 2 | 6 | 1.5 × 10^8^ | No progression | Censored | Alive |
| 38 | 42 | | M | | III (T3N2M0) | | Local, regional nodes | N/MRD | 0 | 1 | 4 | 6 | 2.4 × 10^8^ | PD | 5.5 | Alive |
| 39 | 22 | | M | | III (T3N1M0) | | Lung | N/MRD | 0 | 2 | 2 | 4 | 1.06 × 10^8^ | No progression | Censored | Alive |
| 102 | 57 | | F | | II (T1N1M0) | | Local | N/MRD | 0 | 1 | 2 | 5 | 1.17 × 10^8^ | No progression | Censored | Alive |
| 103 | 48 | | M | | IVA (T4N0M0) | | Not Applicable | N/MRD | 0 | 1 | 1 | 6 | 1.56 × 10^8^ | SD | 35.0 | 70.0 |
| 106 | 66 | | M | | IVB (T2N3bM0) | | Not Applicable | N/MRD | 0 | 1 | 1 | 4 | 1.03 × 10^8^ | No progression | Censored | Alive |

Abbreviations: ARMD, active recurrent/metastatic disease; EBV DNA, Epstein-Barr virus deoxyribonucleic acid; F, female; M, male; N/MRD, no or miminal residual disease; OS, overall survival; PD, progressive disease; PFS, progression free survival; PR, partial response; SD, stable disease.

*Lost to follow-up since 30^th^ December 2009.

**Table S2:** Univariable and Multivariable Analyses for Successful T-cell Expansion

|  | **Univariable analysis** | | | **Multivariable analysis*** | | |
| --- | --- | --- | --- | --- | --- | --- |
| Variable | OR | 95% CI | *P* | OR | 95% CI | *P* |
| Age <55 years | 0.257 | 0.065 to 1.010 | .050 | 0.251 | 0.056 to 0.978 | .035 |
| Sex (male as reference) | 0.999 | 0.998 to 1.002 | .999 | ND | | |
| Baseline plasma EBV DNA <4000 copies/ml | 0.524 | 0.131 to 2.096 | .361 | ND | | |
| Baseline white cell count ≥8.0 x 10^9^/l | 0.152 | 0.033 to 0.693 | .015 | 0.151 | 0.031 to 0.689 | .015 |
| Baseline lymphocyte ≥0.75 x 10^9^/l | 0.308 | 0.091 to 1.039 | .058 | 0.324 | 0.102 to 1.054 | .091 |
| Number of lines of prior chemotherapy | 0.952 | 0.607 to 1.494 | .832 | ND | | |
| N/MRD *v* ARMD | 0.141 | 0.016 to 1.209 | .061 | 0.143 | 0.014 to 0.996 | .020 |

Abbreviations: ARMD, active recurrent/metastatic disease, CI, confidence interval; ND, not done; N/MRD, no or minimal residual disease; OR, odds ratios.

*Only variables found to be significant in the univariable analysis (*P* < .1) were subsequently analyzed in the multivariable analysis.

**Table S3:** Univariable and Multivariable Analyses for Prognostic Factors of PFS

|  | **Univariable analysis** | | | **Multivariable analysis*** | | |
| --- | --- | --- | --- | --- | --- | --- |
| Variable | HR | 95% CI | *P* | HR | 95% CI | *P* |
| Age | 0.993 | 0.952 to 1.037 | .759 | ND | | |
| Sex (male as reference) | 1.079 | 0.251 to 4.635 | .919 | ND | | |
| Number of lines of prior chemotherapy | 1.289 | 0.915 to 1.815 | .147 | ND | | |
| Number of infusions of T-cell therapy <4 | 2.637 | 0.925 to 7.516 | .070 | 2.731 | 0.929 to 7.812- | .115 |
| N/MRD *v* ARMD | 0.095 | 0.021 to 0.420 | .002 | 0.099 | 0.025 to 0.424 | .002 |

Abbreviations: ARMD, active recurrent/metastatic disease; CI, confidence interval; ND, not done; HR, hazard ratio; N/MRD, no or minimal residual disease.
